# Deep Learning-Assisted Prioritization of Candidate Drug Targets in Tumors Using Spatial Multi-Omics

**DOI:** 10.3390/biology15181601

**Published:** 2026-09-11

**Authors:** Yuxian Liu, Xueyan Zhou, Junyuan Zhang, Guochao Liu, Yanni Cao

**Affiliations:** 1 School of Artificial Intelligence, Anhui University of Science & Technology, Huainan 232001, China; yxl@aust.edu.cn (Y.L.); xueyan_zz@163.com (X.Z.); 2024201872@aust.edu.cn (J.Z.); l1747507746@163.com (G.L.); 2 State Key Laboratory of Digital Intelligent Technology for Unmanned Coal Mining, Anhui University of Science & Technology, Huainan 232001, China

**Keywords:** spatial transcriptomics, spatial epigenomics, tumor spatial microenvironment, deep learning, therapy-related spatial neighborhoods, candidate-target prioritization

## Abstract

Background/Objectives: Tumor heterogeneity is reflected in cell composition, molecular states, spatial distribution, and microenvironment interactions. Spatial transcriptomics can map in situ expression and cell states but cannot fully explain upstream regulation, while spatial epigenomics provides complementary evidence such as chromatin accessibility, histone modifications, and deoxyribonucleic acid (DNA) methylation. This review summarizes the applications of spatial multi-omics and deep learning in prioritizing candidate drug targets in tumors. Methods: We reviewed spatial transcriptomics, spatial epigenomics, and combined sequencing technologies, with a focus on how deep learning supports the analysis and integration of spatial multi-omics data for candidate-target prioritization. Results: Deep learning facilitates the detection of abnormal regions, deciphering of cell origins, integration across samples, inference of cell–cell communication, and combination of imaging with omics data. Spatial multi-omics studies provide therapeutic insights into malignant cells, immunosuppression, stromal and vascular remodeling, and invasion and metastasis niches. Based on these applications, candidate targets can be evaluated in a layered manner according to spatial specificity, cell origin, regulatory consistency, reproducibility across patients, functional dependency, disease relevance, and druggability. Conclusions: Spatial multi-omics and deep learning can enhance the systematic and interpretable selection of candidate targets. However, computational associations cannot replace functional validation. Candidate targets still require verification through gene perturbation, drug sensitivity assays, organoids, animal models, and clinical cohorts to confirm their therapeutic potential.

## Simple Summary

Cancer treatment is often complicated by the fact that tumor tissues are not uniform. Different cells occupy distinct locations, communicate with nearby cells, and respond differently to treatment. Spatial multi-omics provides a way to examine these differences while preserving information about where biological changes occur in the tissue. Deep learning can further help identify meaningful patterns within these complex datasets and connect molecular changes with specific cell populations and tissue regions. This review discusses how these approaches may support the discovery and prioritization of candidate drug targets, particularly in regions associated with immune suppression, tissue remodeling, invasion, metastasis, and treatment resistance. However, a computationally identified molecule cannot be a validated therapeutic target without further evidence. Laboratory experiments, drug-response studies, organoid and animal models, and clinical investigations are still required. Together, these methods may provide a more efficient and reliable route from spatial observations to experimentally supported therapeutic targets.

## 1. Introduction

Tumor heterogeneity is expressed not only through molecular-state differences at the single-cell level, but also through the spatial organization of cells, local regulatory states, and patterns of interaction within the microenvironment. Single-cell sequencing has greatly improved the resolution at which the cellular composition and transcriptional states of the tumor microenvironment (TME) can be characterized. Tissue dissociation, however, erases spatial information in situ, making it difficult to recover physical proximity between cells, local communication networks, or the architecture of the tissue [1,2]. This loss of context hampers a systematic understanding of tumor evolution, clonal adaptation, immune escape, treatment tolerance, and variation in drug response [3,4,5]. Spatial omics provides a technical basis for studying spatial heterogeneity in tumors, with spatial transcriptomics widely used for in situ expression profiling. Current research is concerned not only with cellular composition, but also with how cell states, regulatory programs, and microenvironmental components are arranged within tumor tissue. These spatial patterns have been associated with tumor progression and therapeutic response [6,7,8]. Malignant cells coexist with immune, stromal, and vascular components and the extracellular matrix, forming a tumor microenvironment that varies across spatial regions. Differences in this regional organization are associated with variation in prognosis and treatment sensitivity [9,10]. Transcriptional state is only part of the picture. Spatial heterogeneity is also deeply influenced by epigenetic regulation. Deoxyribonucleic acid (DNA) methylation, chromatin accessibility, histone modifications, and enhancer activity can all shape malignant-state transitions, immune evasion, maintenance of stemness, and resistance to therapy. Recent advances in spatial epigenomics have made it possible to examine epigenetic regulatory states in situ. Among these methods, spatial assay for transposase-accessible chromatin using sequencing (spatial ATAC-seq) maps the distribution of chromatin accessibility across tissue and supports the identification of region-specific regulatory elements [11]. Multi-omics co-assays go a step further. Spatial assay for transposase-accessible chromatin and ribonucleic acid (RNA) sequencing (spatial ATAC-RNA-seq) and spatial cleavage under targets and tagmentation and RNA sequencing (spatial CUT&Tag-RNA-seq), for example, simultaneously profile epigenetic regulatory states and gene expression within the same tissue section [12,13]. Spatial DNA methylation-transcriptome co-sequencing methods are also emerging, enabling DNA methylation states and gene-expression patterns to be measured together at defined spatial locations [14]. Collectively, these technologies provide a basis for investigating the regulatory features associated with treatment-relevant spatial patterns. They can help examine why key pathways are active in particular tumor regions and whether candidate molecules are linked to local epigenetic states.

Yet spatial omics data are typically high-dimensional, sparse, noisy, and strongly affected by cross-platform variation. Spatial epigenetic datasets bring further complications, including weak signal, mismatched resolution, and difficult multimodal alignment [15,16]. Scale mismatches also separate histopathology images from spatial transcriptomic and epigenomic maps. As a result, identifying treatment-relevant spatial structures and interpreting their function often requires sophisticated multimodal integration [17]. Deep learning has become an important tool for dissecting the spatial tumor microenvironment through nonlinear feature extraction, graph-based modeling, and multimodal fusion [3,18]. Deep-learning models can integrate histopathology images with spatial transcriptomic and spatial epigenomic data. This integration can help identify treatment-associated spatial structures, trace the cellular origins of candidate targets, and resolve communication axes linked to drug resistance. However, a computational signal alone is not a validated drug target. Confirmation still requires functional perturbation, drug-sensitivity assays, and validation in clinical cohorts [19,20].

**Figure 1:**
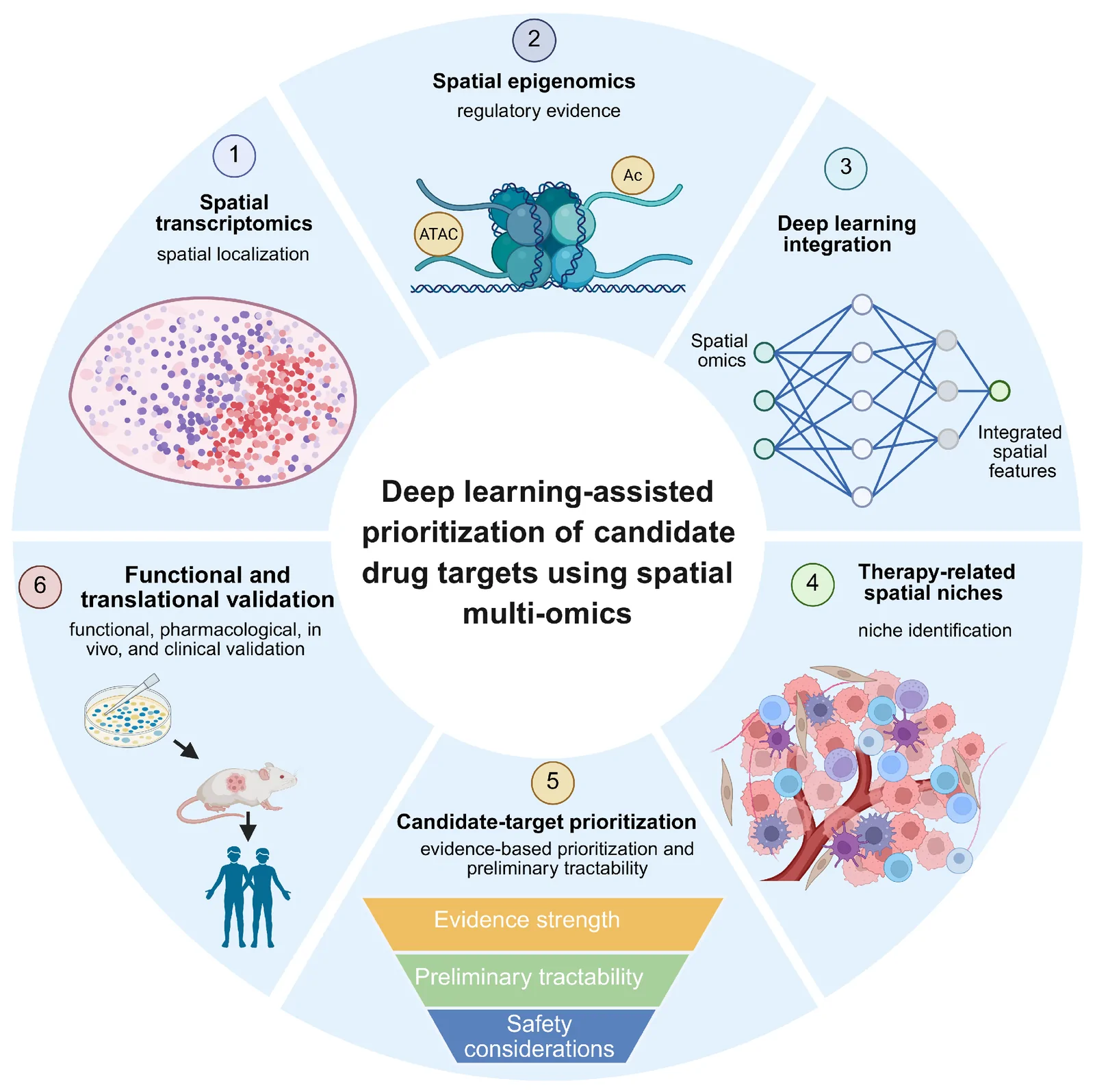
Deep learning-assisted framework for prioritizing candidate drug targets using spatial multi-omics. The workflow proceeds from spatial multi-omics acquisition and deep-learning integration to treatment-related spatial-neighborhood identification, evidence-based candidate-target prioritization, and subsequent functional, pharmacological, and translational validation. Deep learning integrates spatial features but does not independently establish validated drug targets. Note: In Step 1, the color gradient represents differences in the spatial distribution of molecular signals; in Step 2, different colors to distinguish the various components shown in the diagram; in Step 3, colored nodes represent different stages of feature integration within the deep-learning network; and in Step 4, differently colored cells represent distinct cell populations. Other colors are used mainly for visual differentiation and do not convey additional quantitative information. Abbreviations: ATAC, assay for transposase-accessible chromatin; Ac, acetylation.

We argue that spatial multi-omics and deep learning have value beyond characterizing spatial heterogeneity in tumor research. Here, spatial multi-omics broadly encompasses spatially resolved molecular measurements across different omics layers and their joint or integrative analysis. We distinguish joint spatial co-assays or matched-location measurements from computational integration of separately generated spatial, single-cell, or imaging datasets. In the context of candidate-target prioritization, spatial transcriptomics and spatial epigenomics are particularly informative because the former resolves treatment-relevant gene-expression patterns and cellular states, whereas the latter provides complementary information on regulatory states associated with these spatial patterns. By integrating spatial, cellular, and regulatory evidence, these approaches can help identify treatment-relevant spatial microenvironments and support the prioritization of candidate drug targets. Based on this perspective, this review first introduces the technological foundations of spatial transcriptomics and spatial epigenomics. It then summarizes the role of deep learning in spatial-omics analysis. We further discuss treatment-relevant spatial microenvironments from four perspectives: malignant cells, immune suppression, cancer-associated fibroblasts (CAFs) and vascular remodeling, and spatial neighborhoods associated with invasion and metastasis. Building on these themes, we propose a systematic framework for candidate drug-target prioritization. The framework integrates spatial localization, spatial epigenomic regulatory evidence, deep-learning integration, identification of treatment-relevant spatial niches, candidate-target prioritization based on evidence, druggability, safety, and functional and translational validation (Figure 1).

The literature search was conducted primarily using PubMed and Google Scholar in April 2026. Major search terms included “spatial transcriptomics,” “spatial epigenomics,” “spatial multi-omics,” “deep learning,” “tumor microenvironment,” and “drug target.” Original studies relevant to spatial-omics technologies, computational methods, tumor applications, and candidate-target prioritization were prioritized, while recent reviews were used to provide broader context and identify additional primary studies.

## 2. Technical Foundations of Spatial Transcriptomics and Spatial Epigenomics

### 2.1. Spatial Transcriptomic Technologies

Spatial transcriptomics measures gene expression while preserving the location of each signal within tissue. It therefore addresses two major limitations of conventional transcriptomic methods: bulk RNA sequencing (bulk RNA-seq) lacks spatial context, whereas single-cell RNA sequencing removes cells from their native tissue environment [21,22,23,24]. In candidate drug target research, the technology is mainly used to pinpoint the regions in which abnormal genes, signaling pathways, and cell states occur. It can also show whether these abnormalities cluster at invasive fronts, in immunosuppressive zones, around remodeled vasculature, or within treatment-tolerant regions. Spatial transcriptomic methods can be broadly grouped into three classes according to how spatial information is captured and transcripts are read: spatial capture sequencing, imaging-based in situ hybridization, and in situ sequencing. Spatial capture sequencing generally offers broad gene coverage and can survey relatively large tissue areas. This approach is useful for screening spatially differentially expressed genes, dysregulated pathways, and treatment-associated regions. By comparison, imaging-based in situ hybridization provides high spatial or subcellular resolution but often relies on predefined gene panels. Its strength lies in identifying the cellular source of a candidate molecule and mapping its spatial relationship with neighboring cells. In situ sequencing reads RNA molecules or their amplified products directly within intact tissue. The tissue architecture is retained, while transcript information is resolved at the cellular level, allowing local expression patterns of candidate molecules to be validated [25,26,27]. Representative platforms, major applications, and limitations of each class are summarized in Table 1. Overall, spatial transcriptomics can establish the spatial location and cellular origin of candidate molecules. Important challenges remain, including signal mixing, data sparsity, and cross-platform integration [28,29,30,31]. Expression changes alone, however, indicate only that a candidate molecule is associated with a particular spatial state. They rarely reveal the upstream regulatory mechanism. Spatial epigenetic information is therefore needed for a more complete analysis.

### 2.2. Spatial Epigenomic Technologies

Spatial epigenomic technologies can profile regulatory features within intact tissue, including chromatin accessibility, histone modifications, DNA methylation, and three-dimensional genome architecture [44,45,46]. Spatial transcriptomics mainly captures the output of gene expression. By contrast, spatial epigenomics can help assess whether the abnormal expression of a candidate gene or pathway is associated with region-specific regulatory features. Current methods fall into three broad categories. The first comprises spatial epigenomic sequencing techniques. Spatial ATAC-seq, for example, measures chromatin accessibility and supports the identification of putative enhancers and transcription factor binding sites [47]. It has been applied to map chromatin accessibility across tissue sections and to reveal regional differences in epigenetic regulation [11]. Spatial CUT&Tag, meanwhile, charts the regional distribution of histone modifications such as H3K27ac, H3K4me3, and H3K27me3 within tissue [48]. The second category consists of joint epigenome-transcriptome assays. Spatial ATAC-RNA-seq, spatial CUT&Tag-RNA-seq, and spatial DNA methylation-transcriptome co-sequencing can connect epigenetic regulatory states with gene expression at the same spatial location [13,14]. The third category covers imaging-based epigenetic and three-dimensional genome methods, including epigenomic multiplexed error-robust fluorescence in situ hybridization (Epigenomic MERFISH), chromatin tracing, and microscopy-based chromosome conformation capture (Hi-M). Their higher spatial resolution allows selected regulatory loci and chromatin configurations to be examined directly, which is useful when previously nominated regulatory elements or target genes require further validation [45,49,50,51,52,53]. The main classes of spatial epigenomic technologies, together with the features they measure and their applications in candidate drug target research, are summarized in Table 2. Spatial transcriptomic and spatial epigenomic data contribute different types of information to the same biological question. The former shows where treatment-relevant expression patterns, cell states, and functional pathways occur within tumor tissue. The latter can be used to examine whether these patterns are accompanied by local changes in chromatin accessibility, histone modifications, or DNA methylation. When analyzed together, a treatment-relevant region can be linked to putative regulatory elements, transcription factors, and target genes, helping to refine the set of candidate targets.

### 2.3. Challenges in Integrating Spatial Transcriptomic and Spatial Epigenomic Data

Combining these two data types is not straightforward. Spatial transcriptomic datasets are often high-dimensional, sparse, and noisy, and the available platforms vary in spatial resolution, detection throughput, and tissue compatibility. Spot-based sequencing presents an additional problem because one capture spot may contain several cells. The measured expression profile is therefore a mixture of signals from different cellular sources, making direct assignment to individual cell types difficult. Single-cell reference atlases, cell-type deconvolution, and spatial clustering are commonly used to recover local cellular composition and tissue architecture [54,55]. Spatial epigenomic data introduce additional complexity. Signals for chromatin accessibility, histone modifications, and DNA methylation are often even sparser and may differ from transcriptomic data in spatial resolution, noise structure, and biological meaning. Spatial transcriptomics describes expression states. Spatial epigenomics emphasizes the regulatory context behind those states. Integrating the two requires a mapping between epigenetic regulatory states and gene expression without losing their spatial relationships [56,57]. Compounding this problem, tumor tissue is highly heterogeneous in space. Malignant cell states, immune infiltration, stromal remodeling, angiogenesis, and epigenetic programs often change together within local regions. A single feature is therefore rarely sufficient to identify a treatment-relevant spatial structure. Downstream analysis must go beyond denoising, clustering, and estimation of cellular composition. It must also integrate spatial regions, cell states, regulatory programs, cell–cell communication, and treatment response. This need has driven the use of deep learning and related computational approaches. By learning expression patterns, spatial adjacency, and multimodal features from high-dimensional spatial omics data, these methods provide a foundation for identifying spatial structures, comparing samples, and dissecting treatment-relevant microenvironments [58,59,60].

## 3. Deep Learning and Related Computational Methods for Resolving Treatment-Relevant Spatial Biology

### 3.1. Spatial Localization of Treatment-Relevant Molecular Aberrations

Spatial representation learning integrates gene expression with tissue coordinates and neighborhood relationships, allowing high-dimensional spatial data to be represented by a smaller set of informative features. These features can be used to delineate spatial domains and tissue boundaries and to identify region-specific expression patterns. For candidate drug target discovery, however, the first task is usually localization rather than an immediate assessment of druggability. Of interest is where an aberrant gene, pathway, or cell state appears in the tissue, for example, in the tumor core, at the invasive front, in an immune-infiltrated or stroma-rich zone, or within a treatment-tolerant region [61,62,63]. Many methods approach this problem by first constructing a spatial adjacency graph and then modeling molecular profiles together with spatial location. Nearby spots with similar expression patterns can therefore be grouped into histologically meaningful regions. Spatial transcriptomics with an adaptive graph attention auto-encoder (STAGATE), for example, applies graph attention to relationships among neighboring positions and can identify spatial domains and tissue boundaries [61]. Spatially embedded deep representation (SEDR) instead learns a joint low-dimensional representation from expression and spatial features. The resulting representation can support spatial clustering, trajectory inference, and analysis of cellular interactions [63]. GraphST combines graph neural networks with contrastive learning and is used for spatial structure identification and integration across multiple samples [64]. Other related computational methods have been developed for large-scale datasets, spatial expression reconstruction, denoising, and cross-sample integration [62,65,66,67,68]. Such analyses can determine whether a candidate gene or pathway is concentrated in a biologically meaningful compartment, including an invasive front, immunosuppressive niche, remodeled vascular zone, or treatment-tolerant region. This spatial restriction can narrow the search space and indicate which regions warrant further analysis of cellular origin, regulatory mechanisms, and therapeutic tractability.

### 3.2. Cellular Origins of Candidate Therapeutic Signals in Heterogeneous Tumors

Expression measured at a single spatial transcriptomic spot may originate from several cells, which limits direct assignment of an abnormal gene or pathway to a particular cell population. Single-cell reference atlases, spatial expression matrices, and tissue coordinates can therefore be analyzed together through deconvolution or cell-state reconstruction to improve cellular attribution. In candidate target studies, this makes it possible to examine whether a molecule is mainly associated with malignant cells, immune cells, cancer-associated fibroblasts (CAFs), endothelial cells, or other microenvironmental populations. cell2location estimates cellular composition at relatively fine granularity using single-cell references, whereas NLSDeconv and other spatially aware approaches are designed to improve the efficiency and stability of spot-level estimates [54,55,69]. DestVI is not limited to discrete cell-type proportions. It also models continuous variation within individual cell types, which can reveal state heterogeneity among tumor cells, myeloid populations, and lymphocytes across spatial regions [70]. A different strategy is adopted by SpaTopic, which combines spatial clustering with topic-based analysis of cellular composition to describe how different cell populations are organized within complex tumor tissues [71]. ScMalignantFinder provides complementary information by distinguishing malignant cells from nonmalignant components of the microenvironment [72]. The interpretation of a candidate signal depends in part on where that signal originates. Signals associated mainly with malignant cells may be more directly related to tumor-cell proliferation or invasion. Those arising from myeloid cells, CAFs, or endothelial cells may instead be associated with immune suppression, stromal remodeling, or angiogenesis. Resolving these cellular sources also helps avoid attributing a mixed spot-level signal to an inappropriate cell population. This information can then guide subsequent mechanistic analysis and evaluation of possible intervention strategies.

### 3.3. Cross-Sample Integration and Assessment of Cross-Patient Reproducibility

Spatial patterns observed in a single specimen are vulnerable to section position, sampling range, sequencing depth, and patient-specific variation. Even pronounced enrichment on one slide does not establish that a gene or pathway is reproducible across patients or clinical states. Cross-sample integration can facilitate the alignment and comparison of related spatial structures across samples, but integration alone does not demonstrate biological reproducibility. Reproducibility requires that comparable spatial patterns or candidate signals recur across independent patients or clinically relevant sample groups. Several methods tackle this problem from different angles. GraphST jointly models gene expression and spatial neighborhoods to support domain identification and joint analysis across tissue sections [64]. SpatiAlign uses contrastive learning to align spatial expression features from different samples, limiting the influence of technical variation on clustering and differential analysis [68]. BayeSMART incorporates gene expression, spatial neighborhoods, and histopathology images in a unified model to identify comparable regions across samples [73]. Scalable factor analysis for spatial dimension reduction of multi-section spatial transcriptomics (FAST) and stSCI provide additional support through spatial dimension reduction and cross-modal integration, respectively [62,74]. For candidate target discovery, informative comparisons include treatment-sensitive versus resistant tumors, recurrent versus nonrecurrent disease, samples with or without microvascular invasion, paired specimens collected before and after therapy, and primary versus metastatic lesions. Histopathology, marker genes, cellular composition, pathway activity, and clinical outcomes should be evaluated together rather than treated as separate validation layers [75]. Greater priority can be given to candidates that recur in comparable treatment-relevant regions across multiple patients and show consistent associations with poor outcome or resistance. Biological reproducibility across independent patients provides additional support, although it is not sufficient for final validation. Therapeutic relevance also requires consideration of cellular origin, regulatory mechanism, and functional perturbation.

### 3.4. Cell–Cell Communication and Actionable Signaling Axes in the Tumor Microenvironment

Within the spatial tumor microenvironment, treatment-relevant states may be associated with communication among malignant cells, immune populations, CAFs, and endothelial cells. Ligand-receptor co-expression alone does not capture this spatial context. Intercellular distance, neighborhood structure, and downstream transcriptional changes provide additional information on which interactions may be functionally relevant. This allows the analysis to move beyond individual differentially expressed genes and examine signaling relationships together with their regulatory networks. HoloNet, for example, links ligand-receptor relationships between neighboring cells to downstream gene-expression changes and uses this information to prioritize potentially functional communication events [76]. SPIDER instead examines region-specific heterogeneity in communication and incorporates downstream transcription factor activity when evaluating potential function [77]. FineST further combines tissue morphology with spatial molecular information to characterize local cellular interactions in greater detail [78]. Using these approaches, interaction programs can be examined at tumor-immune interfaces, within immunosuppressive or stroma-rich niches, and along invasive fronts. Cell–cell communication (CCC) is also sensitive to ligand-receptor database selection, distance thresholds, and cell-type deconvolution, while transcript-level co-expression does not directly establish protein abundance, ligand secretion, receptor activation, or downstream pathway activity. A ligand-receptor axis may warrant further investigation when it is repeatedly strengthened in resistant or invasive regions, appears across patients, and is accompanied by a coherent downstream transcriptional program. These observations, however, remain inferential. Genetic perturbation, pathway blockade, and drug-sensitivity experiments are still needed before a ligand, receptor, or downstream node can be considered a functional target.

### 3.5. Pathological and Clinical Relevance of Spatial Molecular Phenotypes

Image-omics fusion links tissue morphology in hematoxylin and eosin (H&E)-stained histopathology images with molecular measurements from spatial transcriptomics, spatial proteomics, and related assays. Routine pathology images are widely available and support large cohorts, but they do not directly measure molecular states. Spatial omics offers those measurements in situ, although cost and sample availability limit scale. Deep-learning models that predict spatial expression or phenotype from morphology can therefore extend a treatment-relevant region discovered by spatial omics into broader clinical populations [20,60,79]. THItoGene learns associations between local H&E morphology and spatial gene expression to predict spatial transcriptomic signals [80]. HiST uses a multiscale fusion framework for tumor tissue, combining local image features with spatial expression to reconstruct regional expression patterns [81]. Subsequent work based on transformers, multiscale modeling, and single-cell references has improved fusion by bringing local morphology, tissue architecture, and cell-state information into the same analysis [60,82,83]. Vision-omics foundation models and multimodal pathology foundation models now seek transferable representations from much larger datasets, opening a path toward cross-cancer and cross-task spatial phenotype prediction [20,84]. In candidate target research, image-omics fusion does not prove therapeutic value. Its contribution is different: it tests whether the spatial phenotype associated with a candidate can be recognized in routine pathology. For candidates linked to immune suppression, vascular remodeling, or treatment-tolerant regions, the corresponding phenotype can then be evaluated in independent clinical samples and related to therapeutic outcomes [85]. This expansion of spatial evidence supports patient stratification and identifies cohorts in which functional validation is most informative.

Model validation should be distinguished from the biological reproducibility of candidate spatial patterns. To reduce information leakage, data splitting should preferably be performed at the patient level rather than randomly across spots or cells, and generalizability should be examined in independent cohorts or across platforms when feasible. Robustness and comparisons with simpler baselines should also be considered. Batch and tissue-section effects should also be controlled, and model interpretation should be accompanied by uncertainty estimates where feasible.

To facilitate comparison of the representative methods discussed above, Table 3 summarizes their analytical categories, computational frameworks, main inputs and tasks, integration capabilities, key methodological scope, and code availability.

## 4. Potential Points of Intervention in Treatment-Relevant Spatial Neighborhoods

### 4.1. Spatial Neighborhoods Driven by Malignant Cell States

**Figure 2:**
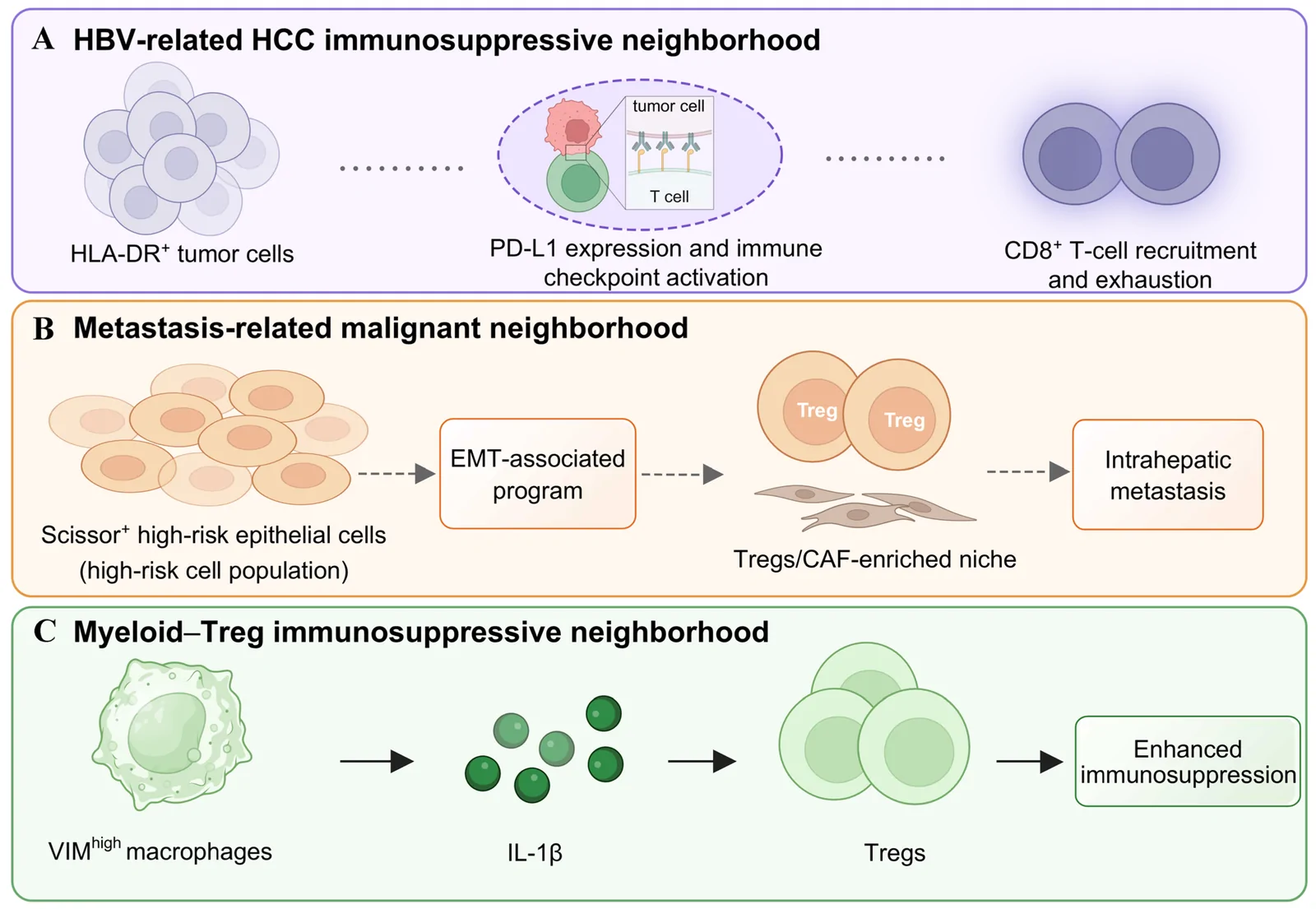
Representative malignant-state and immunosuppressive spatial neighborhoods. (**A**) In HBV-related hepatocellular carcinoma (HCC), HLA-DR^+^ tumor cells are associated with increased PD-L1 expression and immune checkpoint activation, together with increased recruitment and exhaustion of CD8^+^ T cells. (**B**) Scissor^+^ high-risk epithelial cells are accompanied by an EMT-associated program and enrichment of regulatory T cells (Tregs) and cancer-associated fibroblasts (CAFs), and are associated with intrahepatic metastasis. (**C**) VIM^high^ macrophages enhance Treg-mediated immunosuppression through IL-1β, forming a myeloid–Treg immunosuppressive neighborhood. Dotted connectors indicate spatial associations, dashed arrows indicate associative or putative processes, and solid arrows indicate relationships supported by relatively direct functional evidence. Note: In panel A, the pink and green cells in the immune-checkpoint schematic represent tumor cells and T cells, respectively; in panel C, the green circles represent IL-1β signaling molecules. Colors are schematic and do not indicate quantitative differences. Abbreviations: HBV, hepatitis B virus; HLA-DR, human leukocyte antigen-DR; PD-L1, programmed death-ligand 1; CD8, cluster of differentiation 8; EMT, epithelial–mesenchymal transition; VIM, vimentin; IL-1β, interleukin-1 beta.

Spatial neighborhoods driven by malignant cell states are locally organized units in which tumor cells with epithelial–mesenchymal transition (EMT), enhanced stemness, aberrant antigen presentation, or prometastatic features accumulate and establish stable interactions with immune, stromal, and vascular components. Unlike a mere cluster of tumor cells, such a neighborhood is defined by the coupling between the functional state of malignant cells and the surrounding microenvironment. Malignant programs identified by spatial-omics are often concentrated in particular regions of a tumor rather than distributed evenly throughout the tissue [86,87]. These regions can differ in their cellular composition and local interactions, giving rise to distinct functional neighborhoods. In hepatocellular carcinoma (HCC), such spatial organization has been linked to immune escape and metastasis. Chang et al. report that human leukocyte antigen-DR-positive (HLA-DR^+^) tumor cells in hepatitis B virus (HBV) -related HCC are associated with increased programmed death-ligand 1 (PD-L1) expression and immune checkpoint activation, as well as the recruitment and exhaustion of cluster of differentiation 8-positive (CD8^+^) T cells (Figure 2A) [88]. Zhu et al. describe a related but distinct spatial organization. Scissor^+^ epithelial cells are enriched in high-risk populations, together with EMT activation and the accumulation of regulatory T cells (Tregs) and cancer-associated fibroblasts (CAFs). These features form a local structure associated with tumor migration and intrahepatic metastasis (Figure 2B) [89]. Similar spatial patterns also occur in other cancers. In colorectal cancer, regions containing stanniocalcin 2-positive (STC2^+^) malignant cells are associated with EMT, angiogenesis, and immune-cell infiltration [90]. In gastric cancer, a high-stemness (HighStem) subpopulation is restricted to particular regions and shows invasive and prognostically relevant stemness features [91]. In malignant gliomas, spatial transcriptomics further reveals radial glial stem-like tumor cells enriched in neuron-rich invasive niches, with FAM20C implicated in their invasive growth [92]. Despite differences among tumor types, these neighborhoods commonly involve the local enrichment of a specific malignant program together with changes in immune-cell distribution, stromal composition, and vascular signaling. Aberrant antigen presentation, EMT, stemness maintenance, and prometastatic programs may therefore represent potential points for further investigation. Spatial epigenomic data can further examine chromatin accessibility, enhancer activity, and histone modifications within these regions. Such analysis may help assess whether the observed malignant program is associated with a stable regulatory state and identify transcription factors, regulatory elements, and downstream effectors for subsequent validation.

### 4.2. Immunosuppressive Spatial Neighborhoods

Immunosuppressive, treatment-tolerant neighborhoods do not arise simply because more immune cells occupy a region. They are sustained by the stable co-occurrence and cooperative activity of suppressive myeloid cells, Tregs, exhausted T cells, and CAFs within a particular metabolic environment. Through cell–cell communication, inflammatory mediator release, and metabolic reprogramming, these components build a local barrier that continually weakens antitumor immunity. In HCC, the spatial co-occurrence of vimentin-high (VIM^high^) macrophages and Tregs is associated with tumor progression. VIM^high^ macrophages can further enhance the immunosuppressive activity of Tregs through interleukin-1 beta (IL-1β), making the VIM^high^ macrophage-IL-1β-Treg axis a comparatively well-defined candidate signaling axis for therapeutic intervention (Figure 2C) [93]. Evidence from pan-cancer analysis broadens this view. The inflammatory tissue-resident macrophage-enriched niche reported by Wang et al. contains tumor-educated inflammatory tissue-resident macrophages that co-infiltrate with neutrophils and CAFs. Together, they form a cold tumor ecosystem associated with resistance to immune checkpoint blockade (ICB) and poor prognosis [94]. Lactate metabolism and lactylation programs add another layer, as both are closely linked to immunosuppression and resistance to immunotherapy [95,96]. These findings support a network model in which myeloid cell-Treg or myeloid cell-CAF interactions cooperate with metabolic reprogramming to maintain local immune suppression. The IL-1β axis is supported by comparatively strong evidence as a point of intervention. By contrast, myeloid cell-CAF interactions and lactate-associated programs are more appropriately viewed as sources of candidate molecules that still require systematic screening and functional validation.

### 4.3. Spatial Neighborhoods Associated with CAFs and Vascular or Extracellular Matrix Remodeling

**Figure 3:**
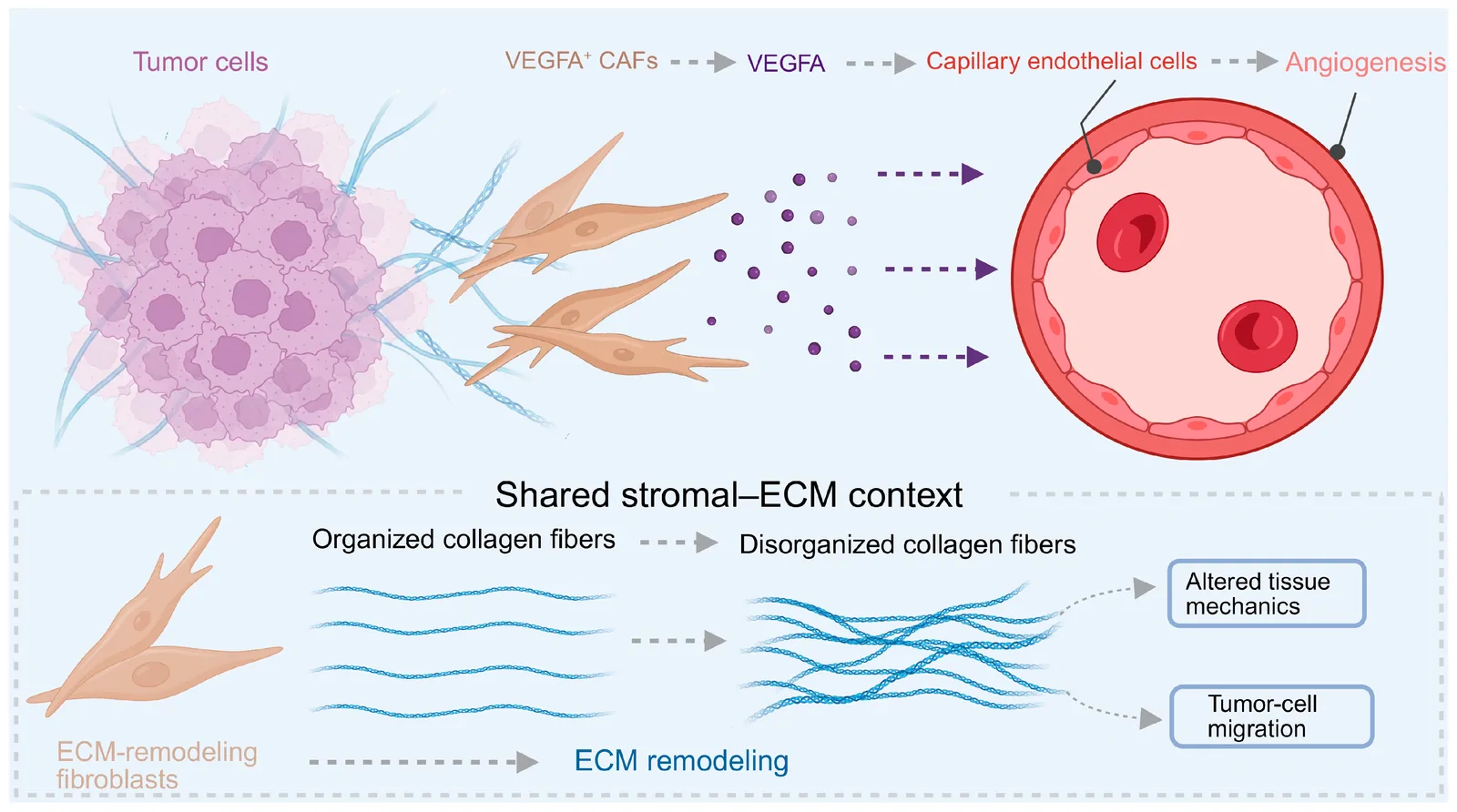
CAF–vascular–ECM remodeling-associated spatial neighborhood. This figure is a conceptual synthesis across studies and tumor types; the depicted components were not necessarily co-measured in the same specimen. Within the tumor interior, VEGFA-positive CAFs are spatially associated with capillary endothelial cells and may promote angiogenesis through VEGFA-related signaling. Within a shared stromal–ECM context, ECM-remodeling fibroblasts are accompanied by a transition from relatively organized to disorganized collagen fibers, which is associated with altered tissue mechanics and tumor-cell migration. Purple dashed arrows indicate VEGFA-related paracrine signaling, whereas gray dashed arrows indicate associative or conceptual processes. Abbreviations: CAFs, cancer-associated fibroblasts; ECM, extracellular matrix; VEGFA, vascular endothelial growth factor A.

Spatial neighborhoods associated with cancer-associated fibroblasts (CAFs) and vascular or extracellular matrix (ECM) remodeling are functional units formed locally by CAFs, endothelial cells, tumor cells, and the ECM. These neighborhoods are linked to angiogenesis, stromal remodeling, tumor invasion, and treatment tolerance [97,98]. These neighborhoods are defined more by coordinated interactions among their components than by the abundance of any single cell type. Such spatial organization is observed across different tumor types. Vascular endothelial growth factor A (VEGFA)-positive CAFs are enriched mainly within the tumor. Their interactions with capillary endothelial cells may promote angiogenesis, and higher abundance of these CAFs is associated with tumor progression and poor prognosis (Figure 3) [99]. In breast cancer, spatial transcriptomic analysis identifies spatially organized FAP^+^ CAF niches, while functional experiments implicate DPP4- and YAP1-dependent mechanisms in the transition from Detox-iCAFs to ECM-myCAFs [100]. Vascular alterations are only part of this organization. In pancreatic ductal adenocarcinoma, ECM-remodeling fibroblasts are enriched in regions associated with EMT activation and tumor progression [98]. Collagen-fiber alignment and the spatial architecture of the stroma can also influence local cell distribution and cellular interactions (Figure 3) [97]. The ECM is therefore more than a structural scaffold. By altering tissue mechanics and cell–cell communication, it may participate in treatment tolerance.

Taken together, VEGFA-associated signaling, DPP4/YAP1-dependent CAF plasticity, and signals of ECM remodeling represent treatment-relevant mechanisms that warrant further investigation.

### 4.4. Spatial Neighborhoods Related to Invasive Fronts, Metastatic Niches, and Treatment Response

Neighborhoods associated with invasive fronts, metastatic niches, and treatment response reflect local interactions among tumor cells, immune populations, endothelial cells, and surrounding normal tissue. Such structures have been linked to invasion, metastasis, recurrence, and therapeutic response. In lung adenocarcinoma, spatial profiling across non-invasive and invasive stages reveals coordinated changes in tumor-cell phenotypes and local immune features, particularly at boundaries between invasive and less malignant regions [101]. In intrahepatic cholangiocarcinoma, C-X-C motif chemokine ligand 5 (CXCL5) and solute carrier family 6 member 14 (SLC6A14) are associated with microvascular invasion and tumor proliferation, as well as immune resistance and poor prognosis [102]. In HCC, INTS6^+^ endothelial cells are enriched in primary tumors and microvascular invasion regions from patients with recurrent disease. Their spatial proximity to tumor cells suggests a possible role in recurrence and metastasis [103]. Treatment-response regions in HCC show distinct immune and vascular features as well. Artificial intelligence-based pathology combined with spatial transcriptomic validation reveals higher expression of immune effector molecules in regions associated with benefit from atezolizumab–bevacizumab therapy [85]. Further studies integrating single-cell transcriptomics with bulk RNA-seq identify immune-active, angiogenesis-driven, and resistant molecular profiles related to therapeutic response [104]. Together, these findings indicate that treatment sensitivity may be shaped jointly by local immune status, vascular features, and tumor-microenvironment interactions. Overall, CXCL5 and SLC6A14 are candidate markers of microvascular invasion. INTS6^+^ endothelial cells and the immune and angiogenic states associated with treatment response offer a different type of evidence: they help identify cellular programs from which candidate targets may be prioritized. These observations remain prioritization clues and require mechanistic validation before they can be treated as therapeutic targets. Across tumor types, HCC and intrahepatic cholangiocarcinoma highlight immunosuppressive, vascular, invasion-, recurrence-, and treatment-response-associated spatial programs, while colorectal and gastric cancers illustrate malignant states associated with EMT or stemness. Malignant glioma illustrates the spatial localization of invasive malignant states, breast cancer highlights spatially organized CAF plasticity, pancreatic ductal adenocarcinoma exemplifies ECM-remodeling stromal niches, and lung adenocarcinoma demonstrates stage-related changes at invasive boundaries.

Overall, malignant cells, immunosuppressive cell populations, CAFs and vascular-associated cells, as well as spatial neighborhoods associated with invasion and metastasis, can provide molecular and cell–cell communication clues that support the prioritization of candidate molecules and signaling axes. However, spatial colocalization or association alone is insufficient to establish therapeutic relevance. Integrating spatial, regulatory, reproducibility, and functional evidence can therefore support the systematic prioritization of candidate targets for subsequent validation.

## 5. Spatial Epigenomics and Candidate Target Discovery

### 5.1. Epigenetic Regulation of Treatment-Relevant Spatial Neighborhoods

The organization of tumor spatial neighborhoods reflects more than cellular composition and transcriptional state. Local epigenetic control is equally important. Changes in chromatin accessibility, enhancer activity, histone modifications, and DNA methylation can contribute to transitions in malignant cell state, immune evasion, angiogenesis, and treatment tolerance. In HCC, epigenetic abnormalities are closely linked to tumor initiation and progression, prognosis, and therapeutic response [105]. Adding spatial epigenetic information may therefore help characterize regulatory features associated with aberrant molecular states at invasive fronts, in immunosuppressive regions, within CAF-rich or vascular-remodeling compartments, and across areas with different treatment responses. Spatial transcriptomics can locate treatment-relevant genes, pathways, and cell states. Spatial epigenomics asks a complementary question: are those expression abnormalities associated with local regulatory states? These sources of regulatory evidence should nevertheless be distinguished. Direct spatial epigenomic assays and joint spatial epigenome–transcriptome assays measure regulatory states in situ or at matched tissue positions, whereas spatially resolved multiregion and non-spatial epigenetic studies provide complementary but not equivalent evidence.

Spatial ATAC-seq identifies region-specific open chromatin, putative enhancers, and transcription factor binding sites [11]. Combined spatial ATAC-RNA-seq and spatial CUT&Tag-RNA-seq link chromatin state or histone modifications to transcriptional output at the same tissue positions [13]. These paired measurements help determine whether abnormal candidate-gene expression in invasive or treatment-tolerant regions coincides with enhancer opening, promoter modification, or altered DNA methylation. As complementary evidence from spatially resolved multiregion profiling, Heide et al. report that most somatic changes in chromatin accessibility are clonally distributed across colorectal tumor regions, although a subset remains spatially restricted [106]. Chromatin-accessibility changes and gene-expression shifts are not always spatially concordant [13]. The difference is informative rather than contradictory. It shows that epigenetic state can provide regulatory evidence that is not apparent from transcription alone. Candidates supported by both spatially restricted expression and a corresponding epigenetic alteration offer a stronger basis for prioritization. The same principle applies to regulatory elements and signaling pathways. Such candidates are better placed to enter subsequent molecular screening and functional validation than signals supported by expression data alone. Studies in glioblastoma, lung cancer, and pancreatic ductal adenocarcinoma also provide complementary regulatory evidence, with spatially resolved or spatially stratified analyses identifying associations between regional tumor states and distinct chromatin-accessibility patterns or regulatory programs [107,108,109]. Spatial epigenomic perturbation, longitudinal, and multiregion designs remain future applications rather than established target-validation strategies.

### 5.2. Spatial Epigenetic Regulation and Target Prioritization

Spatially differentially expressed genes and spatial markers are not synonymous with candidate drug targets. Biomarkers indicate disease state, prognosis, treatment response, or other clinical associations, but they do not necessarily represent functional dependencies or intervention targets. At the screening stage, such signals are referred to here as candidate molecules. Those supported by convergent spatial, regulatory, and reproducibility evidence may be prioritized as candidate drug targets (candidate targets), whereas the term therapeutic target is reserved for targets supported by functional, pharmacological, and safety evidence. Differential expression establishes an association with a particular spatial state, whereas a therapeutic target also requires evidence of causality, tractability, and acceptable safety. Spatial multi-omics cannot provide that proof on its own. It can, however, make candidate screening more reliable by linking a molecule to its location, cellular origin, and regulatory state. Priority should increase when a candidate is enriched at an invasive front, in an area of microvascular invasion, within an immunosuppressive niche, or in a treatment-tolerant region. Concordance between epigenetic and transcriptional changes also matters, as do a defined cellular source, recurrence across samples, disease relevance, potential tractability, and an acceptable safety profile. Public resources such as the Open Targets Platform can integrate target-disease associations, safety information, and druggability evidence into this assessment [110]. Several classes of epigenetic regulators have already emerged as major areas of cancer drug development. These include DNA methyltransferases (DNMTs), histone deacetylases, enhancer of zeste homolog 2 (EZH2), bromodomain and extra-terminal domain proteins, and other chromatin regulators [111].

**Figure 4:**
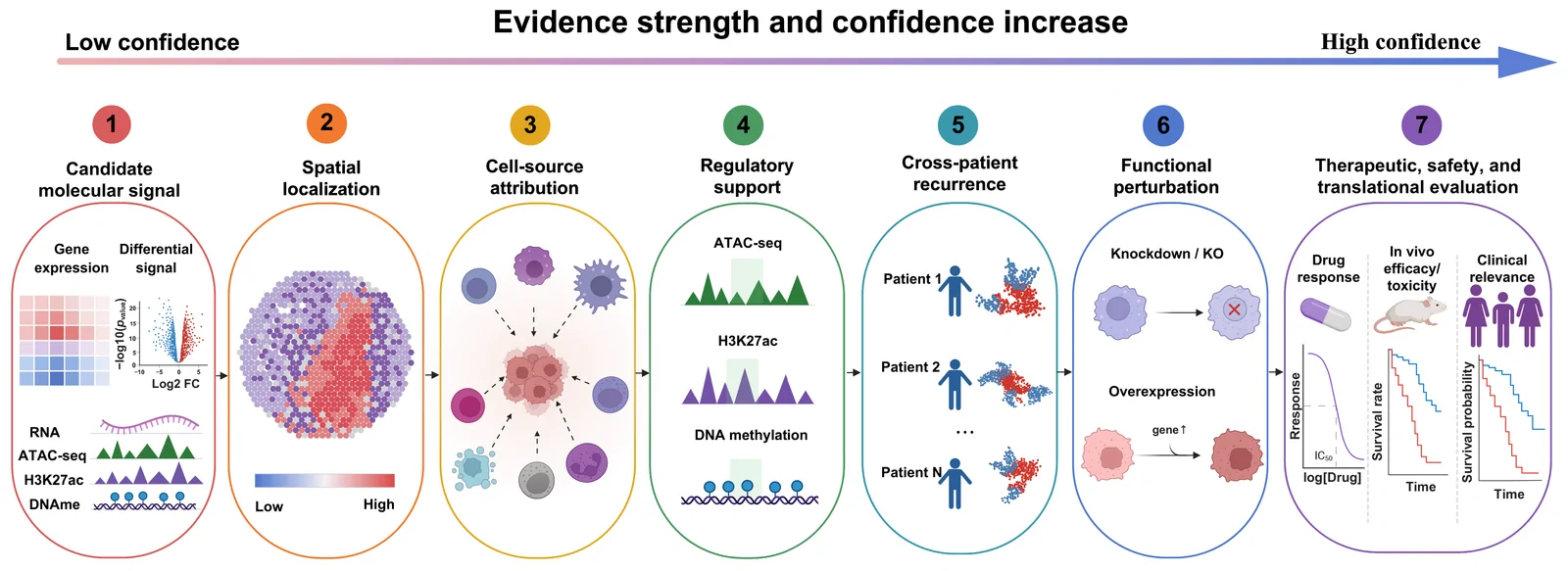
Evidence progression path for screening and validating candidate drug targets. The evidence pathway progresses from candidate molecular signals through spatial localization, confirmation of cell origin, regulatory support, reproducibility across patients, functional perturbation, and therapeutic, safety, and translational evaluation. Spatial localization can also support early safety assessment by comparing candidate signals across tumor regions, invasive margins, and adjacent normal tissue. The arrows indicate a gradual increase in evidence strength and confidence. Note: In Step 3, differently colored cells represent distinct cell populations; in Step 5, the red and blue point clouds schematically distinguish different spatial or molecular patterns across patients; and in Step 7, differently colored response and survival curves represent comparison groups. These colors are schematic and do not indicate quantitative magnitude. Abbreviations: RNA, ribonucleic acid; ATAC-seq, assay for transposase-accessible chromatin using sequencing; FC, fold change; DNAme, DNA methylation; H3K27ac, histone H3 lysine 27 acetylation; KO, knockout; IC_50_, half-maximal inhibitory concentration.

Complementary non-spatial evidence can further support target evaluation. In HCC, regulators such as EZH2 and DNMTs are associated with tumor progression, immune regulation, and response to therapy [112]. For example, combined treatment with DNMT and EZH2 inhibitors activates treatment-relevant molecules in HCC cell lines, suggesting that these epigenetic regulators may offer points of intervention [113]. Complementary multiregion evidence from colorectal cancer also reveals region-specific oncogenic programs. Heide et al. report that aberrant activation of ERBB2, JAK3, interferon regulatory factor/signal transducer and activator of transcription (IRF/STAT)-related pathways, and developmental transcription factor programs can accompany altered chromatin accessibility [106]. By contrast, direct spatial epigenomic evidence from breast cancer is provided by spatial ATAC analysis, which detects open-chromatin signals associated with HER2-positive regions [114]. This provides an example of how spatial chromatin accessibility may support the regulatory interpretation of regional oncogenic signaling. Taken together, these direct and complementary lines of evidence from colorectal cancer, breast cancer, glioblastoma, lung cancer, and pancreatic ductal adenocarcinoma illustrate how spatial and regulatory information can support candidate-target prioritization across tumor types rather than within a single cancer-specific framework. Large-scale clustered regularly interspaced short palindromic repeats (CRISPR)-Cas9 screens provide complementary functional evidence by evaluating tumor-cell dependency and molecular context, which can further inform target prioritization [115]. These forms of evidence should not be weighted equally. Spatial association and differential expression provide screening-level evidence; reproducibility across samples and concordant regulatory support strengthen prioritization, whereas functional perturbation provides stronger evidence of biological dependency. Candidates supported by stronger and convergent evidence can then proceed to druggability, safety, and translational evaluation (Figure 4).

### 5.3. Deep Learning-Driven Integration of Multimodal Evidence

Candidate drug target discovery relies on evidence from spatial location, gene expression, cellular composition, chromatin state, tissue morphology, and clinical outcomes. Deep learning can integrate these sources through graph-based modeling and multimodal feature fusion. Rather than starting with a predefined candidate, the analysis may first locate invasive fronts, immunosuppressive regions, areas of vascular remodeling, or regions with different treatment responses. The next question is which cells contribute to the signal and whether the same pattern recurs across samples. Cellular deconvolution and cross-sample integration can address these issues, while cell–cell communication inference helps characterize the associated signaling network. Regulatory information adds another dimension to this analysis. Chromatin accessibility, histone modifications, DNA methylation, and gene expression can be examined together to relate spatial regions to regulatory elements, transcription factors, candidate target genes, and cell states. Peak-to-gene links, transcription factor motifs, and regulatory network analysis can then be used to examine the upstream regulation of individual candidates [13,111]. Here, deep learning mainly serves to combine these different lines of evidence and reduce the number of candidates requiring further evaluation. A gene restricted to a treatment-tolerant region, for example, may receive greater priority if an open enhancer, a defined cellular source, and recurrence across patients provide additional support. This evidence-integration framework can be applied in different tumor contexts. Spatial multiregion profiling in colorectal cancer has revealed region-specific chromatin-accessibility alterations, whereas spatial ATAC analysis in breast cancer has identified open-chromatin signals associated with HER2-positive regions [106,114]. With multi-sample data, the same analytical workflow can further be used to examine whether comparable spatial programs recur across clinically relevant patient groups, as illustrated by studies of lung adenocarcinoma development, microvascular invasion, HCC recurrence, and treatment-response-associated phenotypes [85,101,102,103]. Candidates supported by consistent spatial localization, cellular attribution, regulatory evidence, and recurrence across patients can therefore be given greater priority. No single layer is sufficient on its own, although agreement across multiple layers can strengthen confidence in a candidate. Model output remains affected by data sparsity, cohort size, platform differences, and interpretability. Histopathological and biological evidence should therefore remain part of the final assessment.

### 5.4. Druggability Assessment and Functional Validation of Candidate Targets

For a candidate to become a viable drug target, its causal function, available modes of intervention, expression in normal tissues, and potential toxicity must be assessed. Spatial profiling preserves the histological relationship among tumor regions, invasive margins, and adjacent normal tissue, allowing candidate expression to be compared across these compartments. When joint spatial transcriptomic and epigenomic measurements are available, gene-expression patterns can also be evaluated together with chromatin accessibility or histone-modification states on the same tissue section [116]. Unlike bulk or single-cell sequencing approaches, which lack native spatial information, spatial profiling enables molecular signals to be compared among tumor regions, invasive margins, and adjacent normal tissue [117]. Such regional selectivity may provide an early indication of potential on-target/off-tumor risk, although it does not establish tolerability and still requires subsequent pharmacological and in vivo safety evaluation.

Cell-surface receptors, secreted factors, enzymes, and epigenetic regulators may be amenable to antibodies, small-molecule inhibitors, or other therapeutic modalities. Transcription factors, enhancers, and other difficult-to-drug nodes may instead be approached through their upstream regulators or downstream effector pathways. Validation can begin with mechanistic studies in vitro. CRISPR interference or activation, RNA interference, and gene overexpression can test whether a candidate gene or regulatory element affects tumor-cell proliferation, invasion, immune evasion, or treatment tolerance [118]. Spatial CRISPR methods extend this analysis while preserving tumor architecture. They can reveal how a genetic perturbation changes local cellular composition, immune state, and the tumor microenvironment, moving the evidence from spatial association toward causal testing [119]. Drug-sensitivity assays, cell co-culture systems, and patient-derived organoids provide the next level of evidence. Patient-derived organoids retain aspects of the molecular features and drug responses of the original tumor, offering an individualized platform for testing candidate targets and related therapeutic strategies [120].

Animal models are then needed to examine antitumor activity, toxicity, and effects on the microenvironment in vivo. Independent clinical cohorts are equally important. Spatial transcriptomics, in situ hybridization, spatial proteomics, and multiplex immunofluorescence can assess the recurrence and spatial localization of candidate targets across independent clinical samples, while histopathological image analysis can evaluate the associated tissue phenotype. Protein-level validation should assess not only abundance and spatial localization but, where relevant, functional activity using phosphorylation- or enzyme-activity-sensitive measurements, because protein abundance alone does not establish target or pathway activity. These approaches also allow the spatial phenotype to be related to prognosis, recurrence, metastasis, and therapeutic response. Only after functional perturbation, pharmacological testing, animal studies, and validation in clinical samples can a candidate regulatory signal be advanced as a therapeutically meaningful drug target. Spatial multi-omics locates treatment-related abnormalities and relates them to associated epigenetic regulatory states. Deep learning integrates that information with cellular origin, cross-sample recurrence, functional dependency, and clinical relevance. Together, the two approaches support a continuous workflow from candidate discovery and prioritization to druggability assessment and functional validation.

## 6. Challenges and Future Perspectives

Despite the potential of spatial multi-omics and deep learning for candidate-target prioritization, several gaps still limit their translation. Spatial associations and computational predictions do not establish causal dependency, and candidate signals may show limited reproducibility across patients, cancer types, and technical platforms. Assessment of normal tissue toxicity, drug delivery feasibility, and therapeutic windows also remains relatively insufficient. The cross-cancer examples discussed above suggest that this framework is not restricted to a single tumor type and can be extended to comparative analyses across patient cohorts when sufficiently matched spatial and clinical data are available.

Future work should move beyond spatial association and place greater emphasis on causal and translational evidence. Spatial multi-omics can be combined with perturbation experiments, longitudinal sampling, and multiregion sampling to examine how local cell states, spatial neighborhoods, and cell communication change after candidate-target intervention. Candidate-target prioritization also needs a more transparent basis, with the strength and uncertainty of different evidence types considered separately. In addition, normal tissue expression, potential toxicity, drug delivery feasibility, and therapeutic windows should be evaluated earlier rather than after candidate selection. This may help shift spatial multi-omics-driven candidate-target prioritization from correlation-based screening toward mechanistic and translational validation.

## 7. Conclusions

Spatial transcriptomics and spatial epigenomics provide complementary evidence for candidate-target prioritization, including spatial localization, cell-of-origin information, and potential regulatory states. Deep learning can further connect these data with tissue morphology, gene expression, and clinical information. Based on the evidence discussed in this review, we recommend that candidate targets be prioritized through convergent evidence rather than any single molecular signal or computational score. Greater priority should be given to candidates with spatial specificity, a clear cellular origin, concordant regulatory support and expression changes, reproducibility across patients, functional dependency, disease relevance, and potential druggability. Spatial multi-omics and deep learning are therefore more suitable for candidate discovery and evidence-based prioritization than for direct target confirmation. Final validation still requires functional perturbation, drug sensitivity testing, organoid and animal studies, independent clinical cohorts, and further assessment of safety and therapeutic windows.

## Figures and Tables

**Table 1:** Common spatial transcriptomic technologies and their roles in candidate drug target research.

Technology Class	Representative Platforms	Main Role in Candidate Drug Target Research	Key Limitations
Spatial capture sequencing	10x Visium [32,33], Slide-seq [34], Stereo-seq [35], DBiT-seq [36]	Screens large tissue areas for aberrantly expressed genes, treatment-related pathways, and spatially restricted molecular states	Some platforms produce mixed multicellular signals. Resolution and sample compatibility vary across platforms.
Imaging-based in situ hybridization	Xenium [37], MERFISH [38], seqFISH^+^ [39], CosMx SMI [40]	Validates the cellular source, spatial boundaries, and neighboring-cell relationships of candidate molecules at single-cell or subcellular resolution	Some methods rely on predefined gene panels. Detection breadth, cost, and throughput must be balanced.
In situ sequencing	STARmap [41], FISSEQ [42], BaristaSeq [43]	Reads RNA molecules or barcode sequences within intact tissue to localize and validate candidate molecules at high resolution	Workflows remain complex, and both detection efficiency and tissue compatibility require further optimization.

Abbreviations: Stereo-seq, SpaTial Enhanced REsolution Omics-sequencing; DBiT-seq, deterministic barcoding in tissue for spatial omics sequencing; MERFISH, multiplexed error-robust fluorescence in situ hybridization; seqFISH+, sequential fluorescence in situ hybridization plus; SMI, Spatial Molecular Imager; STARmap, spatially resolved transcript amplicon readout mapping; FISSEQ, fluorescent in situ sequencing; RNA, ribonucleic acid.

**Table 2:** Spatial epigenomic technologies and their roles in candidate drug target research.

Technology Class	Representative Methods	Principal Features Measured	Role in Candidate Drug Target Research
Spatial epigenomic sequencing	Spatial ATAC-seq, spatial CUT&Tag	Chromatin accessibility, promoter and enhancer activity, and histone modifications	Assesses whether candidate genes or pathways are associated with region-specific regulatory features and identifies putative transcription factors and regulatory elements
Joint epigenome-transcriptome sequencing	Spatial ATAC-RNA-seq, spatial CUT&Tag-RNA-seq, spatial DNA methylation-transcriptome co-sequencing	Epigenetic regulatory states and gene expression	Links regulatory abnormalities with transcriptional output at the same spatial location, thereby increasing confidence in candidate target selection
Imaging-based epigenetic and three-dimensional genome methods	Epigenomic MERFISH, chromatin tracing, Hi-M	Specific epigenetic loci, chromatin conformation, and spatial relationships among regulatory regions	Provides high-resolution validation of candidate regulatory elements, target genes, and long-range regulatory interactions

Abbreviations: ATAC-seq, assay for transposase-accessible chromatin using sequencing; CUT&Tag, cleavage under targets and tagmentation; DNA, deoxyribonucleic acid; RNA, ribonucleic acid; MERFISH, multiplexed error-robust fluorescence in situ hybridization; Hi-M, microscopy-based chromosome conformation capture.

**Table 3:** Representative deep-learning and related computational methods for treatment-relevant spatial analysis.

Category	Method and Computational Framework	Main Input	Main Task and Relevance to Candidate-Target Prioritization	Integration Capability	Key Limitation/Scope	Code Availability
Spatial localization	STAGATE [61]: Graph attention auto-encoder	Expression + spatial graph	Spatial-domain identification; localizes candidate-associated spatial regions	Multi-section ST	ST-focused; localization does not establish therapeutic relevance	GitHub: zhanglabtools/STAGATE
	SEDR [63]: Deep autoencoder + variational graph autoencoder	Expression + spatial information	Clustering, trajectory analysis, and denoising; identifies spatially restricted candidate-associated patterns	Multi-batch ST	Inferred patterns require downstream validation	GitHub: JinmiaoChenLab/SEDR
	GraphST [64]: GNN + contrastive learning	spatial graph; optional scRNA-seq	Clustering, integration, and deconvolution; supports spatial and cellular interpretation of candidate signals	Multi-sample ST; scRNA-seq + ST	Cross-modal integration is not direct molecular spatial multi-omics	GitHub: JinmiaoChenLab/GraphST
Cellular attribution	Cell2location [54]: Bayesian model	Single-cell reference + ST	Cell-type mapping; attributes candidate signals to specific cell populations	sc/snRNA-seq + ST	Reference-dependent	GitHub: BayraktarLab/cell2location
	NLSDeconv [69]: Non-negative least squares	Reference profiles + ST	Cell-type deconvolution; supports cell-of-origin assignment of candidate signals	Reference + ST	Reference-based estimation	GitHub: tinachentc/NLSDeconv
	DestVI [70]: Variational inference	scRNA-seq + ST	Deconvolution and cell-state mapping; resolves candidate-associated cellular states	scRNA-seq + ST	Model-based estimates; computational requirements may limit use	GitHub: romain-lopez/DestVI-reproducibility; scvi-tools
	SpaTopic [71]: Topic modeling/statistical learning	scRNA-seq + SRT	Spatial-domain analysis and deconvolution; links candidate signals to local cellular composition	scRNA-seq + SRT	Transcriptomics-focused; not direct spatial molecular multi-omics	GitHub: compbioNJU/SpaTopic
	ScMalignantFinder [72]: Logistic-regression classifier	scRNA-seq; ST for spatial application	Malignant-cell and malignant-region identification; attributes candidate signals to malignant cells	scRNA-seq + ST	Classification does not demonstrate therapeutic dependency	GitHub: jonyyqn/scMalignantFinder
Cross-sample integration	SpatiAlign [68]: Unsupervised contrastive learning	Multi-sample SRT + spatial locations	Alignment and batch correction; supports assessment of recurrence of candidate-associated spatial patterns	Multi-sample SRT	Integration does not by itself demonstrate biological reproducibility	GitHub: STOmics/Spatialign
	BayeSMART [73]: Bayesian statistical model	Multi-sample SRT + histology	Spatial-domain clustering; supports comparison of candidate-associated regions across samples	Multi-sample SRT + histology	Histology-assisted integration is not direct molecular spatial multi-omics	GitHub: yg2485/BayeSMART
	FAST [62]: Probabilistic factor analysis	Multi-section SRT	Spatial dimension reduction; facilitates comparison of candidate-associated patterns across sections or samples	Multi-section SRT	Primarily dimension reduction; downstream biological interpretation is required	GitHub: feiyoung/ProFAST
	StSCI [74]: Multi-task learning	scRNA-seq + ST	Integration, deconvolution, and reconstruction; supports cellular interpretation of candidate signals across datasets	scRNA-seq + ST	Transcriptomic integration rather than direct joint spatial multi-omics	GitHub: hannshu/stSCI
Cell–cell communication	HoloNet [76]: Multi-view graph learning	ST + LR pairs + downstream expression	CCC inference with downstream-expression support; prioritizes candidate signaling axes	Multiple ST-derived information layers	Communication remains inferential and requires experimental validation	GitHub: lhc17/HoloNet
	SPIDER [77]: Probabilistic models + SOM	ST + LR + downstream information	Spatially variable LRI inference; prioritizes region-specific candidate signaling interactions	Multiple ST-derived information layers	LRI inference requires functional validation	GitHub: deepomicslab/SPIDER
	FineST [78]: Contrastive multimodal learning	Histology + ST	High-resolution imputation and CCC analysis; refines candidate-associated local interactions	Histology + ST	Includes imputed rather than directly measured expression	GitHub: StatBiomed/FineST
Image–omics integration	THItoGene [80]: Dynamic convolution + capsule network	Histology + ST	Spatial-expression prediction; extends candidate-associated molecular phenotypes to histology	Histology + ST	Predicted expression requires downstream validation	GitHub: yrjia1015/THItoGene
	HiST [81]: Multiscale convolutional deep learning	Histology + ST	Spatial-expression reconstruction; supports pathology-based extension of candidate-associated spatial phenotypes	Histology + ST	Reconstructed expression requires validation	GitHub: Yelab2020/HiST
	MISO [60]: Multiscale deep learning	Histology + ST	Spatial-expression prediction and multiscale morphology–omics integration; supports evaluation of candidate-associated spatial phenotypes from histology	Histology + ST	Predicted molecular information requires external and downstream validation	GitHub: owkin/miso_code
	SciSt [82]: Single-cell reference-informed deep learning	Histology + scRNA-seq reference + ST	Spatial-expression prediction; incorporates cellular reference information into candidate-associated morphology–expression analysis	Histology + non-spatial single-cell reference + ST	Depends on segmentation and reference-data quality; predicted expression requires validation	GitHub: liyixin12139/SciSt
	OmiCLIP/Loki [20]: Visual–omics foundation model	Histology + ST; auxiliary molecular references	Cross-modal representation and spatial-expression prediction; supports transferable analysis of candidate-associated phenotypes	Histology + ST and auxiliary reference data	Predicted molecular states require downstream validation	GitHub: GuangyuWangLab2021/Loki

Note: Integration capability refers to multi-sample, cross-modal, or multimodal analysis. Integration of scRNA-seq with spatial transcriptomics or histology with spatial transcriptomics does not by itself represent direct joint measurement of multiple spatial molecular-omics layers. Accordingly, computational integration using non-spatial single-cell references or histology is distinguished from direct integration of jointly profiled spatial molecular modalities. These methods can support candidate-target prioritization through spatial localization, cellular attribution, cross-sample comparison, signaling inference, or image–omics integration, but they do not independently establish validated therapeutic targets. Abbreviations: CCC, cell–cell communication; GNN, graph neural network; LR, ligand-receptor; LRI, ligand-receptor interaction; scRNA-seq, single-cell RNA sequencing; snRNA-seq, single-nucleus RNA sequencing; SOM, self-organizing map; ST, spatial transcriptomics; SRT, spatially resolved transcriptomics.

## Data Availability

No new data were created or analyzed in this study. Data sharing is not applicable to this article.

## Abbreviations

The following abbreviations and key terms are used in this manuscript:

**Full Term**	**Abbreviation and/or Brief Definition**
Ribonucleic acid	RNA—A nucleic acid molecule that conveys genetic information and serves as the primary molecular readout in transcriptomic analyses.
Deoxyribonucleic acid	DNA—The genetic material that stores hereditary information and provides the molecular basis for genomic and epigenomic analyses.
Assay for transposase-accessible chromatin	ATAC—An assay used to profile chromatin accessibility and identify open chromatin and putative regulatory elements.
Assay for transposase-accessible chromatin using sequencing	ATAC-seq—Profiles chromatin accessibility and supports identification of open chromatin and putative regulatory elements.
Assay for transposase-accessible chromatin and RNA sequencing	ATAC-RNA-seq—Joint profiling of chromatin accessibility and gene expression; spatial implementations preserve tissue-location information.
Biological reproducibility	Recurrence of comparable spatial patterns or candidate signals across independent patients or clinically relevant sample groups.
Bulk RNA sequencing	bulk RNA-seq—Measures average transcript abundance across a bulk tissue sample without preserving native spatial information.
Cancer-associated fibroblasts	CAFs—Stromal fibroblasts associated with tumor progression, extracellular-matrix remodeling, immune regulation, and treatment-related spatial states.
Candidate drug target	Candidate target—A candidate supported by convergent spatial, regulatory, reproducibility, and related evidence and prioritized for subsequent functional and therapeutic evaluation.
Candidate molecule	A screening-level molecular signal identified from spatial, expression, or other association-based analyses that has not yet been prioritized as a drug target.
Cell–cell communication	CCC—Signaling relationships among cell populations; inferred ligand–receptor interactions alone do not establish functional communication.
Cleavage under targets and tagmentation	CUT&Tag—Profiles protein-associated chromatin features, including histone modifications.
Cleavage under targets and tagmentation and RNA sequencing	CUT&Tag-RNA-seq—Joint profiling of chromatin-associated regulatory states and gene expression; spatial implementations preserve tissue location.
Cluster of differentiation 8	CD8—A cell-surface molecule commonly associated with cytotoxic T-cell populations.
Clustered regularly interspaced short palindromic repeats	CRISPR—A genome-editing and perturbation system used for functional assessment of candidate genes or regulatory elements.
Computational integration	Computational combination of separately generated spatial, single-cell, imaging, or other datasets; it is not equivalent to direct joint measurement of multiple spatial molecular-omics layers.
C-X-C motif chemokine ligand 5	CXCL5—A chemokine discussed in this review in relation to microvascular invasion and treatment-relevant spatial states.
DNA methyltransferases	DNMTs—Enzymes involved in DNA methylation and epigenetic regulation.
Druggability	The potential of a candidate target to be modulated using an available or feasible therapeutic modality or intervention strategy.
Enhancer of zeste homolog 2	EZH2—An epigenetic regulator involved in histone methylation and chromatin regulation.
Epithelial–mesenchymal transition	EMT—A cellular-state transition associated with invasive and metastatic phenotypes.
Extracellular matrix	ECM—Structural and signaling components surrounding cells that contribute to tissue organization, mechanics, and cell–cell interactions.
Hematoxylin and eosin	H&E—Routine histopathological staining used to evaluate tissue morphology and architecture.
Hepatocellular carcinoma	HCC—Primary liver cancer discussed as one of several representative tumor types in this review.
Human leukocyte antigen-DR	HLA-DR—A major histocompatibility complex class II molecule involved in antigen presentation.
Immune checkpoint blockade	ICB—Immunotherapy targeting inhibitory immune-checkpoint pathways to enhance antitumor immune responses.
Hepatitis B virus	HBV—A virus associated with chronic liver disease and hepatocellular carcinoma.
Programmed death-ligand 1	PD-L1—An immune checkpoint ligand involved in the regulation of T-cell responses and tumor immune evasion.
Interferon regulatory factor/signal transducer and activator of transcription	IRF/STAT—Regulatory programs involving interferon regulatory factors and signal transducer and activator of transcription proteins.
Interleukin-1 beta	IL-1β—A pro-inflammatory cytokine involved in immune-cell communication and regulation.
Joint spatial multi-omics	Joint measurement of multiple molecular-omics layers within the same tissue section or at matched spatial locations.
Multiplexed error-robust fluorescence in situ hybridization	MERFISH—An imaging-based spatial transcriptomic method that enables high-resolution detection of predefined RNA targets.
Single-cell RNA sequencing	scRNA-seq—Transcriptomic profiling at single-cell resolution that can provide reference information for cell-type or cell-state attribution in spatial analysis.
Spatial epigenomics	Spatially resolved profiling of regulatory features such as chromatin accessibility, histone modifications, and DNA methylation, providing regulatory information associated with spatial molecular patterns.
Spatial multi-omics	Spatially resolved molecular measurements across different omics layers and their joint or integrative analysis.
Spatial transcriptomics	ST—Spatially resolved measurement of gene expression while preserving tissue-location information.
Therapeutic target	A target supported by stronger functional, pharmacological, and safety evidence beyond computational or association-based prioritization.
Treatment-relevant spatial neighborhood	A spatially organized local tissue region characterized by coordinated malignant, immune, stromal, vascular, or regulatory features associated with disease progression or treatment response.
Tumor microenvironment	TME—The local cellular and extracellular environment surrounding tumor cells, including immune, stromal, vascular, and extracellular-matrix components.
